# Correction: *RUNX1A* isoform is overexpressed in acute myeloid leukemia and is associated with *FLT3* internal tandem duplications

**DOI:** 10.1038/s41417-025-00982-w

**Published:** 2025-11-03

**Authors:** Cosimo Cumbo, Francesco Tarantini, Elisa Parciante, Luisa Anelli, Antonella Zagaria, Giuseppina Biondi, Mariano Francesco Caratozzolo, Paola Orsini, Nicoletta Coccaro, Giuseppina Tota, Immacolata Redavid, Maria Rosa Conserva, Angela Minervini, Crescenzio Francesco Minervini, Vito Pier Gagliardi, Mario Delia, Flaviana Marzano, Claudia Telegrafo, Sharon Natasha Cox, Annalisa Natalicchio, Bachir Balech, Apollonia Tullo, Mattia Gentile, Francesco Giorgino, Giorgina Specchia, Pellegrino Musto, Francesco Albano

**Affiliations:** 1 Department of Precision and Regenerative Medicine and Ionian Area (DiMePRe-J) - Hematology and Stem Cell Transplantation Unit - University of Bari “Aldo Moro”, Bari, Italy; 2 Department of Precision and Regenerative Medicine and Ionian Area (DiMePRe-J), Section of Internal Medicine, Endocrinology, Andrology and Metabolic Diseases, University of Bari “Aldo Moro”, Bari, Italy; 3 Institute of Biomembranes, Bioenergetics and Molecular Biotechnologies (IBIOM) - National Research Council (CNR), Bari, Italy; 4 Department of Human Reproductive Medicine, Medical Genetics Unit, ASL Bari, Bari, Italy; 5 Department of Biosciences, Biotechnology and Environment, University of Bari “Aldo Moro”, Bari, Italy; 6 School of Medicine, University of Bari “Aldo Moro”, Bari, Italy

**Keywords:** Biomarkers, Leukaemia, Cancer genomics

Correction to: *Cancer Gene Therapy* 10.1038/s41417-025-00939-z, published online 15 July 2025

The article ‘RUNX1A isoform is overexpressed in acute myeloid leukemia and is associated with FLT3 internal tandem duplications’, written by Cosimo Cumbo 1,7, Francesco Tarantini1,7, Elisa Parciante1,7, Luisa Anelli1, Antonella Zagaria1, Giuseppina Biondi2, Mariano Francesco Caratozzolo 3, Paola Orsini4, Nicoletta Coccaro1, Giuseppina Tota1, Immacolata Redavid1, Maria Rosa Conserva1, Angela Minervini1, Crescenzio Francesco Minervini1, Vito Pier Gagliardi1, Mario Delia1, Flaviana Marzano3, Claudia Telegrafo3, Sharon Natasha Cox5, Annalisa Natalicchio2, Bachir Balech3, Apollonia Tullo3, Mattia Gentile4, Francesco Giorgino2, Giorgina Specchia6, Pellegrino Musto1 and Francesco Albano 1,*, was originally published under exclusive license to The Author(s), under exclusive licence to Springer Nature America, Inc. 2025. As a result of the subsequent decision to publish the article under the open access model, the article’s copyright notice was changed on 25. October 2025 to © The Author(s), 2025 and the article is now distributed under a Creative Commons Attribution 4.0 International License, which permits use, sharing, adaptation, distribution and reproduction in any medium or format, as long as you give appropriate credit to the original author(s) and the source, provide a link to the Creative Commons licence, and indicate if changes were made.

The images or other third-party material in this article are included in the article’s Creative Commons license, unless indicated otherwise in a credit line to the material. If material is not included in the article’s Creative Commons license and your intended use is not permitted by statutory regulation or exceeds the permitted use, you will need to obtain permission directly from the copyright holder.

To view a copy of this license, visit http://creativecommons.org/licenses/by/4.0/. Open access funding provided by Università degli Studi di Roma Tor Vergata within the CRUI-CARE Agreement.

